# Molecular identification of *Plasmodium* species in symptomatic children of Democratic Republic of Congo

**DOI:** 10.1186/s12936-018-2480-5

**Published:** 2018-09-20

**Authors:** Hugo Kavunga-Membo, Gillon Ilombe, Justin Masumu, Junior Matangila, Joël Imponge, Emile Manzambi, Francis Wastenga, Dieudonné Mumba Ngoyi, Jean-Pierre Van Geetruyden, Jean Jacques Muyembe

**Affiliations:** 1 Institut National de Recherche Biomédicale (INRB), P.O Box 1197, Kinshasa 1, Kinshasa, Democratic Republic of the Congo; 2 Université Pédagogique National (UPN), Kinshasa, Democratic Republic of the Congo; 3 Laboratoire Vétérinaire de Kinshasa, Kinshasa, Democratic Republic of the Congo; 4 Universite de Kinshasa (UNIKIN), Kinshasa, Democratic Republic of the Congo; 5 Université d’Anvers, Antwerp, Belgium

**Keywords:** *Plasmodium falciparum*, *Plasmodium malariae*, *Plasmodium vivax*, *Plasmodium ovale*, *Plasmodium knowlesi*, Kinshasa, North Kivu, Democratic Republic of Congo

## Abstract

**Background:**

Worldwide, the highest malaria mortality is due to *Plasmodium falciparum* infection. However, other species of *Plasmodium* (*Plasmodium vivax*, *Plasmodium ovale*, *Plasmodium malariae*, and *Plasmodium knowlesi*) can also cause malaria. Therefore, accurate identification of malaria species is crucial for patient management and epidemiological surveillance. This study aimed to determine the different *Plasmodium* species causing malaria in children under 5 years old in two provinces (Kinshasa and North Kivu) of the Democratic Republic of Congo (DRC).

**Methods:**

From October to December 2015, a health-facility based cross-sectional study was conducted in General Reference Hospitals in Kinshasa and North Kivu. Four hundred and seven blood samples were collected from febrile children aged ≤ 5 years. Nested polymerase chain reaction assays were performed for *Plasmodium* species identification.

**Results:**

Out of 407 children, 142 (34.9%) were infected with *Plasmodium* spp. and *P. falciparum* was the most prevalent species (99.2%). Among those infected children, 124 had a mono infection with *P. falciparum* and one with *P. malariae*. Mixed infections with *P. falciparum*/*P. malariae* and *P. falciparum*/*P. vivax* were observed in 6 (1.5%) and 8 (2.0%) children, respectively. The prevalence of infection was higher in females (64.8%) than in males (35.2%), p < 0.001. The age-specific distribution of infection showed that children of less than 2 years old were less infected (18.4%) compared to those aged above 2 years (81.6%), p < 0.001.

**Conclusion:**

Although this study showed clearly that the most prevalent species identified was *P. falciparum*, the findings demonstrate the existence of non-*falciparum* malaria, especially *P. malariae* and *P. vivax* among children aged ≤ 5 years living both Kinshasa and North Kivu Provinces in DRC.

## Background

Malaria is the major cause of morbidity and mortality in many tropical and sub-tropical countries, with half of the world’s population at risk [1]. Malaria is caused by parasites of the genus *Plasmodium* [2], among which five species are known to infect humans: *Plasmodium falciparum*, *Plasmodium malariae*, *Plasmodium ovale*, *Plasmodium vivax* and *Plasmodium knowlesi*.

*Plasmodium falciparum* is responsible for major morbidity and for mortality due to severe clinically forms [3]. However, non-falciparum malaria is also found in sub-Saharan Africa, representing < 10% of the cases [4]. Despite control measures, malaria-related morbidity and mortality remain significantly high in many developing countries, preventing many school children from attending school due to illness, diminishing their capacity to realise their full potential and continues to have a severe socioeconomic impact on their populations [5, 6]. *Plasmodium vivax* has been reported from time to time in populations in sub-Saharan Africa, especially when polymerase chain reaction (PCR) assays have been used [7, 8], but vivax malaria is generally considered to be uncommon in sub-Saharan Africa, because a high proportion of the population has Duffy negative red cells, which cannot be invaded by *P. vivax*. *Plasmodium ovale* and *P. malariae* are relatively common in many parts of East and West Africa causing a significant proportion of febrile malaria episodes [9] and asymptomatic infections. In the context of globalization, implying movement of population from a place to another, and environment changes, the epidemiology of *Plasmodium* species is subject to change. Since anti-malarial treatments depend of the parasite causing the disease, the monitoring of the distribution of *Plasmodium* species is essential [10, 11]. In the Democratic Republic of Congo (DRC), malaria is one of the leading causes of death, especially in children of less than 5 years of age and *P. falciparum* represent the main species responsible of morbidity and mortality [12]. Others *Plasmodium* species have been reported in DRC, but these observations were based on microscopy, not always appropriate for species distinction.

The aim of the present study was to determine *Plasmodium* species distribution using sensitive methods, such as molecular techniques, in samples coming from two DRC provinces (Kinshasa and North Kivu).

## Methods

### Study sites

**Fig. 1:**
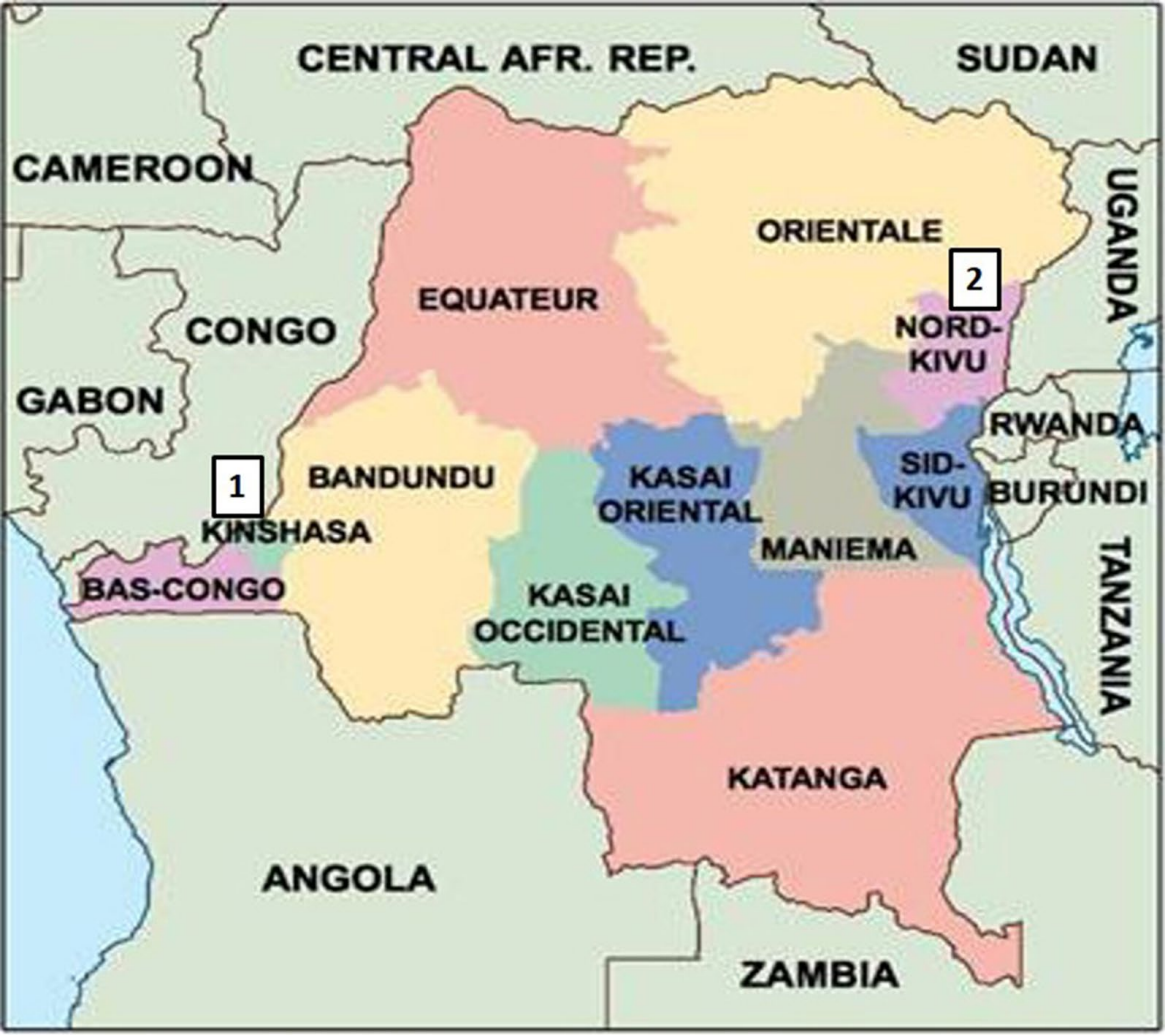
Map of Democratic Republic of Congo. 1: Kinshasa Province, 2: North Kivu Province

Kinshasa Province is an urban area in the western DRC, which hosts up 15% of the DRC’s 79 million people [13]. Kinshasa has a tropical wet and dry climate. Its lengthy rainy season spans from October through May, with a relatively short dry season, between June and September (Fig. 1). Malaria prevalence is diverse given the geographical make-up of the city, with densely populated areas separated by large semi-rural areas, and with some of the areas completely rural in nature [12]. Recently, the prevalence of malaria was fund to be around 11.9% in children 6–59 months, and as high as 31.7% in semi-rural areas of the city [12].

North Kivu province is located in the eastern DRC. This province is mountainous, including a large portion of the Virunga National Park (Fig. 1). Some parts are forested, others are mostly savanna with occasional trees. The soil in the south is generally rich and fertile. The climate is temperate and wet in the mountains, with temperatures between 3 and 18 °C. Since 1992, the population has suffered from ongoing violent conflict between different rebel groups and government troops. Between 2009 and 2012, the cumulative number of malaria cases has never exceeded 40,000 per year, but in 2013 there was an increase of malaria cases among children of less than 5 years of age [14].

### Study design

From October to December 2015, a cross sectional study was conducted in Kinshasa and North Kivu provinces in the DRC. Febrile children aged ≤ 5 years, seeking care for suspected malaria at the selected General Reference Hospitals, were systematically enrolled, once written consent was obtained and subject eligibility assessed.

### Sample collection

Blood samples were obtained from finger prick, collected on filter paper (Whatman No 1), dried, stored in individual plastic bags with desiccant and, then transferred to the National Institute for Biomedical Research in Kinshasa for molecular work.

### DNA extraction

DNA extraction from the blood spots on filter paper was carried out according to the protocol of Musapa et al. [15]. Briefly, the blood spot samples of 5 mm diameter size were soaked in 1 ml of phosphate-buffered saline (PBS), incubated for 30 min at room temperature in a 1.5-ml tube, and centrifuged at 7000 rpm. After centrifugation the supernatant was discarded and 200 µl of chelex 1% were added. The mixture was first incubated at 56 °C at 30 min, then at 100 °C for 8 min and centrifuged at 7000 rpm for 5 min. The supernatant was directly transferred into a 1.5 ml tube and kept at − 80 °C for further analysis.

### Polymerase chain reaction (PCR) for *Plasmodium* species identification

Nested PCR was performed as a two stage procedures: The first round of DNA amplification was performed using rPLU5 and rPLU6 primers (Table 1) in order to determine the *Plasmodium* genus. The PCR mixture had a total volume of 25 µl, containing 1X buffer, 200 μM dNTPs, 10 µM of each primer, 2.5 mM MgCl_2_, 1 unit of *Taq* DNA polymerase (Invitrogen, Carlsbad, CA) and 2 μl of DNA solution. The thermoprofile consisted of an initial denaturation step at 94 °C for 5 min, followed by 25 cycles at 94 °C for 45 s, 58 °C for 45 s, and 72 °C for 1 min, and a final extension step at 72 °C for 5 min. Amplicons were subjected to electrophoresis for approximately 40 min at 100 V on standard 2% agarose gels. The expected size was 1200 bp (Table 2).

**Table 1:** Names and sequences of primers

Names of primers	Sequences of primers
rPLU5	5′-CCTGTTGTTGCCTTAAACTTC-3′
rPLU6	5′-TTAAAATTGTTGCAGTTAAAACG-3′
rFAL-F	5′-CTTTTGAGAGGTTTTGTTACTTTGAGTAA-3′
rFAL-R	5′-TATTCCATGCTGTAGTATTCAAACAAAA-3′
rOVA-F	5′-TTTTGAAGAATACATTAGGATACAATTAATG-3′
rOVA-R	5′-CATCGTTCCTCTAAGAAGCTTTACCCT-3′
rVIV-F	5′-ACGCTTCTAGCTTAATCCACATAACT-3′
rVIV-R	5′-ATTTACTCAAAGTAACAAGGACTTCCAAGC-3′
rMAL-F	5′-ATAACATAGTTGTACGTTAAGAATAACCGC-3′
rMAL-R	5′-AAAATTCCCATGCATAAAAAATTATACAAA-3′

**Table 2:** Showing the expected band size for *Plasmodium* Genus and Species

Genus or/species	Name of primers	Expected band size (bp)
*Plasmodium* Genus	rPLU5 and rPLU6	1200
*Plasmodium falciparum*	rFAL-F and rFAL-R	205
*Plasmodium vivax*	rVIV-F and rVIV-R	120
*Plasmodium malariae*	rMAL-F and rMAL-R	140
*Plasmodium ovale*	rOVA-F and rOVA-R	800

### Nested PCR

All positive samples from the first PCR were subjected to the second PCR round. One microlitre aliquot from the product of the first round was used as template for *Plasmodium* species-specific fragment amplification using four pairs of primers (rFAL-F and rFAL-R, rOVA-F and rOVA-R, rVIV-F and rVIV-R, rMAL-F and rMAL-R) (Table 1). The PCR mixture and cycling conditions were exactly the same as described in the first PCR round, but the number of cycle was increased to 30. PCR products were run for 40 min on a 2% agarose gel, then stained with a 0.5 µg/ml ethidium bromide solution and visualized under ultraviolet transilluminator. The expected size of the PCR products are described in Table 2.

### Data management and statistical analyses

Data were entered and analysed using Epi-info 7 and SPSS 21 (Chicago USA), respectively. Descriptive statistics were used to summarize data (frequencies, mean, and median). Chi square was utilized for proportion comparison. Ninety five (95) % confidence interval and p value set at 0.05 were used for significance.

### Ethical considerations

The study was approved by Institutional Ethics Committee of the School of Public Health of Kinshasa/DRC. Written signed informed consent was obtained from the parents or legal tutors of each child before his/her enrollment in the study. Malaria positive cases were treated according to national malaria diagnosis and treatment guidelines [16].

## Results

### General characteristics

In total, 407 children aged ≤ 5 years old with the clinical suspicion of malaria were enrolled. Of the 407 selected children, 206 (50.6%) were female with sex ratio of 0.765 (Table 3). Median age was 3 years (range 1.7–5.0).

**Table 3:** Patient’s distribution by province, age, sex and type of infection

Variables	Global		Province				p value
			Kinshasa		Nord Kivu		
	n	% (IC95%)	n	% (IC95%)	N	% (IC95%)	
Number total of participants	407		180		227		
Sex							0.765
Female	206	50.6 (45.7–55.5)	93	51.7 (44.3–59)	113	49.8 (43.256.3)	
Male	201	49.4 (44.5–54.3)	87	48.3 (40.9–55.7)	114	50.2 (43.2–56.8)	
Median age years (IQR)	407	3.0 (1.7–5.0)	180	5.0 (3.3–5.2)	227	2 (1–3)	0.000^*^
Parasitology							
PAnPalu							0.012^*^
Positive	142	34.9 (30.2–39.5)	75	41.7 (34.4–48.9)	67	29.5 (23.5–35.5)	
Negative	265	65.1 (60.5–69.7)	105	58.3 (51.1–65.6)	160	70.5 (64.5–76.5)	
Mono-infection							
*P*. *falciparum*							0.009^*^
Positive	141	34.6 (30.0–39.3)	75	41.7 (34.4–48.9)	66	29.1 (23.1–35.0)	
Negative	266	65.5 (60.7–69.9)	105	58.3 (51.1–65.6)	161	70.6 (64.9–76.9)	
*P*. *vivax*							0.146
Positive	8	1.9 (0.06–0.3)	6	3.3 (0.07–0.6)	2	0.9 (0.3–2.1)	
Negative	399	98.0 (96.7–99.4)	174	96.7 (94–99.3)	225	99.1 (97.9–100)	
*P*. *malariae*							0.019^*^
Positive	7	1.7 (0.4–2.9)	0	0	7	3.1 (0.8–5.3)	
Negative	400	98.3 (97–99.5)	180	100	220	96.9 (94.7–99.2)	
Mixed-infections							0.017^*^
*P. falciparum* + *P*. *vivax*	8	2.0 (0.6–3.3)	6	3.3 (0.69–5.9)	2	0.8 (0.3–2.1)	
*P. falciparum* + *P*. *malariae*	6	1.5 (0.2–2.2)	0	0	6	2.6 (0.5–4.7)	

### Overall prevalence of *Plasmodium*

**Fig. 2:**
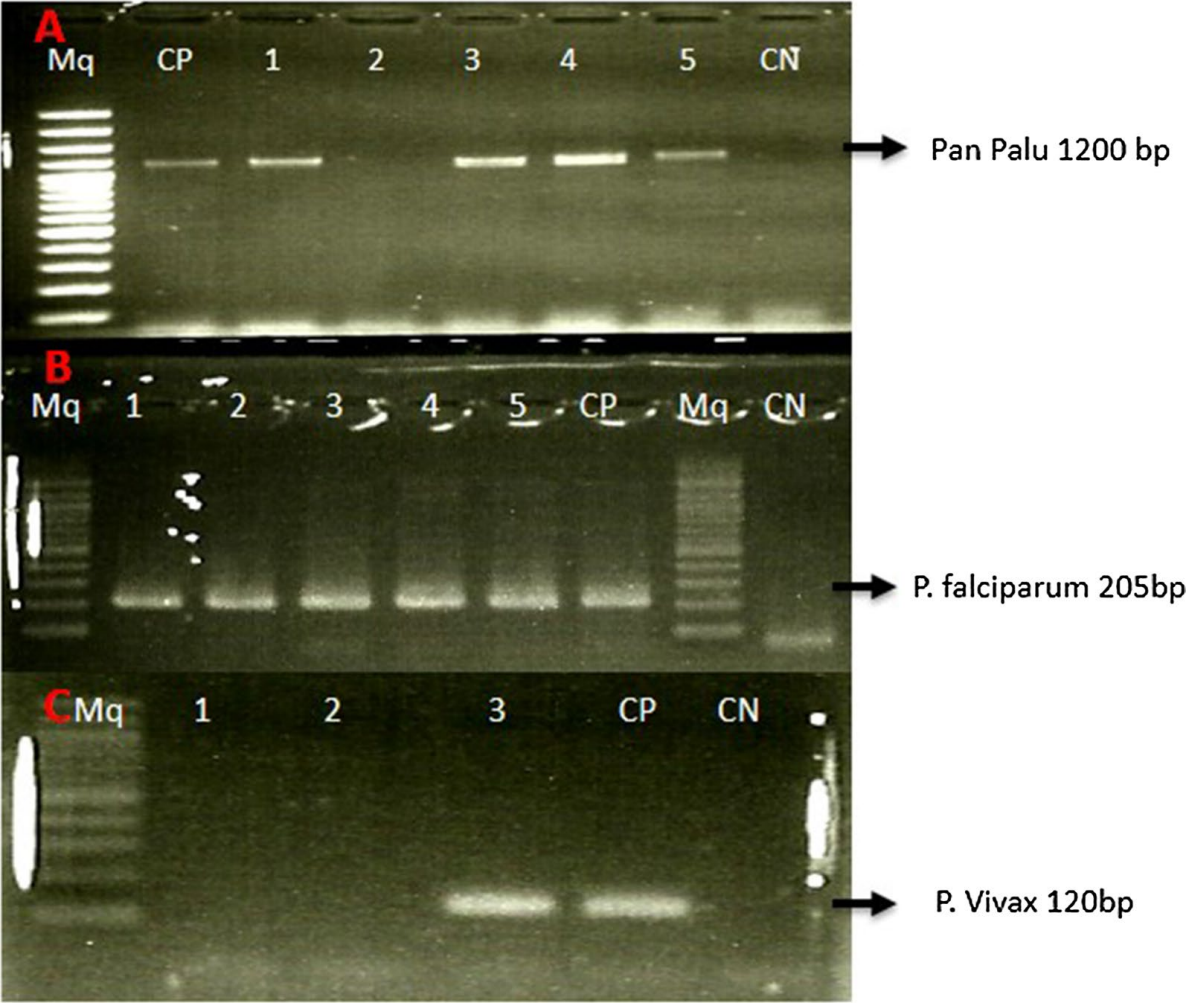
A 2% agarose gel electrophoresis showing DNA amplification of *Plasmodium* Samples with various Primers. **a** A 2% agarose gel electrophoresis showing DNA amplification of *Plasmodium* genus using rPLU5 and rPLU6 primers. Lane 1, 3, 4, 5 represent positive isolates, lane 2 represents negative isolate, lane CP represents positive control, lane CN represents negative control and lane Mq represents Molecular weight maker 100 bp plus DNA ladder. **b** A 2% agarose gel electrophoresis showing DNA amplification of *Plasmodium falciparum* using rFAL-R and rFAL-F primers. Lane from 1 to 5 represent positive isolates, lane CP represents positive control, lane CN represents negative control and lane Mq represents Molecular weight maker 100 bp plus DNA ladder. **c** A 2% agarose gel electrophoresis showing DNA amplification of *Plasmodium vivax* using rVIV-R and rVIV-F primers. Lane from 1 to 2 represent negative isolates, lane 3 represents positive isolate, lane CP represents positive control, lane CN represents negative control and lane Mq represents Molecular weight maker 100 bp plus DNA ladder

Out of 407 samples analysed, 142 (34.9%) were positive for *Plasmodium* sp. infection. *Plasmodium falciparum* was the most prevalent species and was found in 141 (99.2%) of the positive samples (Fig. 2). Mono infection of *P. falciparum* was found in 124 samples and one of *P. malariae*. Mixed infections with *P. falciparum* and *P. malariae* were observed in 6 samples, while 8 samples were positive for *P. falciparum* and *P. vivax*. The prevalence of infection was higher in females (64.8%) than in males (35.2%) (p < 0.001). The age-specific distribution of infection in children showed that children of less than 2 years old were less infected (18.4%) compared to those aged above 2 years (81.6%), p < 0.001.

### Prevalence of *Plasmodium* province-by-province

Kinshasa province reported 75 (41.7%) positive cases for *Plasmodium* spp. infection including *P. falciparum* and *P. vivax* cases. Among the positive cases, the study revealed 6 (3.3%*)* mixed infection cases of *P. falciparum* and *P. vivax* (Table 3).

In the North Kivu Province, 67 (29%) cases were positive for *Plasmodium* spp. infection including *P. falciparum*, *P. vivax* and *P. malariae* cases with 2 (0.8%) mixed infection cases between *P. falciparum* + *P. vivax* and 6 (2.6%) mixed infection cases of *P*. *falciparum *+ *P. malariae* (Table 3).

## Discussion

Malaria is still a major public health concern in most endemic areas in sub-Saharan Africa, especially in the DRC, where 14 million cases have been reported by the WHO in 2014 [1]. *Plasmodium falciparum* is known to induce more severe infections [3], but once detected *P. falciparum* is more susceptible to anti-malarial drugs than other species [4]. Thus, the identification of epidemiological pattern of the disease in malaria-endemic area is important for intervention programmes and treatment purposes.

In the past, species characterization of *Plasmodium* could be achieved using the parasitological methods whereby different species of *Plasmodium* were discriminated by the morphology of the parasite and its position in the red blood cell. However, misinterpretations cannot be ruled out. Thanks to the development of molecular techniques, PCR with species-specific primers can be used with more accuracy [17]. These techniques were successfully used in various areas [17, 18] and, for the first time, have been applied in Kinshasa and North Kivu provinces of the DRC, using field samples collected from children.

As expected results from study revealed a high prevalence of *P. falciparum* as previously reported in Tanzania and DRC [19, 20]. However the study has also shown the presence of non-falciparum *Plasmodium* species circulating in children during the same period and in the same area, including the co-circulation of *P. malariae* and *P. vivax*. In general, the distribution of *P. malariae* coincides with that of *P. falciparum* in malaria endemic areas of Africa, explaining the high frequency of mixed infections of *P. malariae* with *P. falciparum* infections [20]. In the present study such co-infections with *P. falciparum* and *P. malariae* have been found in 1.5% of samples.

The current study revealed also the presence of *P. vivax* infecting children from DRC (Fig. 2). This is the first report on the occurrence of this *Plasmodium* species in DRC using molecular tools. Although the public health importance of *P. vivax* is overshadowed by *P. falciparum*, it has been shown that there is a strong association of *P. vivax* with certain severe malaria symptoms and it accounts for about 390 million clinical cases annually in Asia and South America [21, 22]. Indeed *P. vivax* is rare in Central Africa but it is known to be endemic in some populations of Sudan, Somalia, Ethiopia, Djibouti [22, 23]. Thus its presence in DRC cannot be surprising especially with the displacement of people in this part of Africa. The circulation of people from Asia that is greatly increasing in the eastern part of the DRC can also increase the prevalence of this *Plasmodium* species in this area.

Most research on Duffy antigen in malaria cases over the past 20 years has showed that people with erythrocyte Duffy blood group-negative people were unable to develop *P. vivax* malaria [24, 25]. However, with the availability of molecular diagnostics, observations of *P. vivax* PCR-positive, Duffy-negative individuals have been made [26]. This is a proof that *P. vivax* has broken through its dependence on the Duffy antigen for establishing human blood-stage infection and disease. In DRC limited studies have been conducted on erythrocyte polymorphisms associated with malaria susceptibility. So, further study will be necessary to investigate the Duffy antigen status among our *P. vivax* infection to elucidate its role in DRC Malaria cases.

This presence of *P. malariae* and *P. vivax* among children living in DRC should make the determination of the infecting *Plasmodium* species important in terms of treatment because certain *Plasmodium* infections can cause rapidly progressive severe illness or death while the other infection of *Plasmodium* are less likely to cause severe manifestations or have different drug resistance patterns in differing geographic regions [4, 10, 23].

## Conclusion

Although the highest number of malaria cases involving *P. falciparum*, the current study documented the presence of the non-falciparum malaria parasites in Kinshasa and North Kivu Provinces of the DRC. These findings suggest further surveys in other settings of DRC and a regular surveillance of these non-*falciparum sp* prevalence.

## References

[1] Hay SI, Okiro EA, Gething PW, Patil AP, Tatem AJ, Guerra CA (2010). Estimating the global clinical burden of *Plasmodium falciparum* malaria in 2007. PLoS Med..

[2] Miller LH, Ackerman HC, Su XZ, Wellems TE (2013). Malaria biology and disease pathogenesis: insights for new treatments. Nat Med.

[3] Ciceron L, Jaureguiberry G, Gay F, Danis M (1999). Development of a Plasmodium PCR for monitoring efficacy of antimalarial treatment. J Clin Microbiol.

[4] Williams J, Njie F, Cairns M, Bojang K, Coulibaly SO, Kayentao K (2016). Non-falciparum malaria infections in pregnant women in West Africa. Malar J..

[5] WHO (1999). World Health Organization report 1999—making a differences.

[6] Carter R, Mendis K (2002). Evolutionary and historical aspects of the burden of malaria. Clin Microbiol Rev.

[7] Culleton R, Ndounga M, Zeyrek FY, Coban C, Casimiro N, Takeo S (2009). Evidence for transmission of *Plasmodium vivax* in the Republic of the Congo, West Central Africa. Clin Infect Dis..

[8] Fru-Cho J, Bumah VV, Safeuku I, Nkuo-Akenji T, Titanji VPK, Haldar K (2014). Molecular typing revelas substantial *Plasmodium vivax* infection in asymptomatic adults in a rural area of Cameroon. Malar J..

[9] Roucher C, Rogier C, Sokhna C, Tall A, Trape JF (2014). A 20-year longitudinal study of *Plasmodium ovale* and *Plasmodium malariae* prevalence and morbidity in a West African population. PLoS ONE.

[10] Doderer-Lang C, Atchade PS, Meckert L, Haar E, Perrotey S, Filisetti D (2014). The ears of the African elephant; unexpected high seroprevalence of *Plasmodium ovale* and *Plasmodium malariae* in healthy populations in Western Africa. Malar J..

[11] WHO. World Malaria Report 2015. Geneva, World Health Organization, 2015.

[12] Ferrari G, Ntuku HM, Schmidlin S, Diboulo E, Tshefu AK, Lengeler C (2016). A malaria risk map of Kinshasa, Democratic Republic of Congo. Malar J..

[13] CIA. DRC: The World Fact Book; 2016. https://www.cia.gov/library/publications/the-world-factbook/geos/cg.html. Accessed 10 Mar 2017.

[14] Ministry of Health (2015). Plan National de Développent Sanitaire 2011–2015 et données de surveillance 2015.

[15] Musapa M, Kumwenda T, Mkulama M, Chishimba S, Norris DE, Thuma PE (2013). A simple Chelex protocol for DNA extraction from *Anopheles* spp. J Vis Exp..

[16] Programme National de Lutte Contre le Paludisme, Democratic Republic of Congo (PNLPDRC). Rapport Annuel des Activités de Lutte contre le Paludisme 2010

[17] WHO. Guidelines for the treatment of malaria, 3rd edn. Geneva: World Health Organization; 2015. http://www.who.int/malaria/publications/atoz/9789241549127/en/. Accessed 10 Sept 2015.

[18] Abanyie FA, Arguin PM, Gutman J (2011). State of malaria diagnostic testing at clinical laboratories in the United States, 2010: a nationwide survey. Malar J.

[19] Kim MJ, Jung BK, Chai JY, Eom KS, Yong TS, Min DY (2015). High malaria prevalence among schoolchildren on Kome Island, Tanzania. Korean J Parasitol..

[20] Mvumbi DM, Bobanga TL, Melin P, De Mol P, Kayembe JM, Situakibanza HN (2016). High prevalence of *Plasmodium falciparum* infection in asymptomatic individuals from the Democratic Republic of the Congo. Malar Res Treat..

[21] Ketema T, Bacha K (2013). *Plasmodium vivax* associated severe malaria complications among children in some malaria endemic areas of Ethiopia. BMC Public Health..

[22] Tiono AB, Diarra A, Sanon S, Nébié I, Konaté AT, Pagnoni F, Sirima SB (2013). Low specificity of a malaria rapid diagnostic test during an integrated community case management trial. Infect Dis Ther..

[23] Deressa W, Ali A, Enqusellassie F (2003). Self-treatment of malaria in rural communities, Butajira, southern Ethiopia. Bull World Health Organ.

[24] Boyd MF, Stratman-Thomas WK (1933). Studies on benign tertian malaria. 4. On the refractoriness of Negroes to inoculation with *Plasmodium vivax*. Am J Epidemiol.

[25] O’Leary PA (1927). Treatment of neurosyphilis by malaria: report on the three years’ observation of the first one hundred patients treated. J Am Med Assoc.

[26] Ménard D, Barnadas C, Bouchier C, Henry-Halldin C, Gray LR, Ratsimbasoa A (2010). *Plasmodium vivax* clinical malaria is commonly observed in Duffy-negative Malagasy people. Proc Natl Acad Sci USA.

